# Prediction of high-pressure phases of Weyl semimetal NbAs and NbP

**DOI:** 10.1038/s41598-017-13610-x

**Published:** 2017-10-16

**Authors:** Jingyun Zhang, Cuihong Yang, Weifeng Rao, Jian Hao, Yinwei Li

**Affiliations:** 1 Jiangsu Key Laboratory for Optoelectronic Detection of Atmosphere and Ocean, Nanjing University of Information Science & Technology, Nanjing, 210044 China; 2 Department of Physics, School of Physics & Optoelectronic Engineering, Nanjing University of Information Science & Technology, Nanjing, 210044 China; 3 Department of Materials Physics, and IEMM, Nanjing University of Information Science & Technology, Nanjing, 210044 China; 4 School of Physics and Electronic Engineering, Jiangsu Normal University, Xuzhou, 221116 China

## Abstract

As the first known Weyl semimetals, binary compounds including TaP, TaAs, NbAs, and NbP have received worldwide interest. This work explored the phase behaviours of NbAs and NbP under high pressure up to 200 GPa via first-principles calculations combined with intelligent particle swarm optimization. Upon compression, a new phase of NbAs with *P-6m2* symmetry appeared above 23 GPa and remained stable until 38 GPa, whereupon a monoclinic structure with space group *P2*_1_ */c* became more energetically favourable. This lasted until 73 GPa, when a *Pm-3m* phase followed. Surprisingly, NbP underwent a single phase transition around 63.5 GPa to a new phase with *Cmcm* symmetry that was completely distinct from the structures shown by TaAs-class compounds such as NbAs and TaAs. All these newly pressure-stabilized structures were dynamically stable at both high and ambient pressure. Electronic band structure calculations revealed a transition from semimetal to metal under high pressure. This work is meaningful and fundamental for future studies and applications of TaAs-class Weyl semimetals under compression or extreme conditions.

## Introduction

Weyl semimetals are crystals hosting both Weyl fermions with definite chirality (low-energy electronic excitations) and Fermi arcs caused by topological surface states. They are a newly discovered class of topologically non-trivial materials beyond topological insulators. Many interesting physical characteristics have been reported, such as extremely large magnetoresistance^1–3^ and unusual transport mobility^4^ stemming from the chiral anomaly^1^, and thus these semimetals represent a new era in condensed matter physics. It is interesting to note that the Weyl nodes defined as the crossing points between two non-degenerate bands can be observed in the negative magnetoresistance measurements^5^. Furthermore, the transport evidence of the intriguing surface states can be realized through investigating Aharonov–Bohm oscillations^6^. First-principles simulations firstly predicted non-centrosymmetric transition-metal monophosphides to be Weyl semimetals^7,8^. Consequent photoemission spectroscopy of TaAs single crystals directly observed the Weyl semimetal state (Fermi arcs on the surface, together with Weyl fermion cones and Weyl nodes in the bulk)^9,10^. Independent angle-resolved photoemission experiments also confirmed TaAs as a Weyl semimetal^11^. NbAs was later identified experimentally as a Weyl semimetal by soft X-ray and ultraviolet photoemission spectroscopy^12^. Further works reported the Weyl state in other members of the same family such as TaP^13^ and NbP^14^. These first-known Weyl semimetals (all TaAs-class compounds) each have twelve pairs of Weyl nodes, which are entirely stoichiometric and nonmagnetic in the whole Brillouin zone. These promising discoveries suggest there are many exotic topological properties to be investigated in TaAs-class Weyl semimetals^15–19^, which might lead to potential applications in electronics, optoelectronics, and quantum computing.

Pressure is commonly used to alter the chemical environment to induce novel properties and produce new compounds. Combined theoretical simulation and synchrotron experiments have found a new Weyl semimetal phase of TaAs with *P-6m2* symmetry and only six pairs of Weyl nodes that appeared above 14 GPa, followed by a *P2*_*1*_ */c* phase at around 34 GPa^20^. However, high-pressure studies of NbAs and NbP have been lacking up to date. This work reports a comprehensive and systematic study of new phases in NbAs and NbP under compression up to 200 GPa, and analyses their band structures and electron localization function in detail. A transition sequence of *I4*_*1*_ *md* → *P-6m2* → *P2*_*1*_ */c* → *Pm-3m* was observed in NbAs, while a single phase transition of *I4*_*1*_ *md* → *Cmcm* took place in NbP. Our work is meaningful and fundamental to future theoretical and experimental investigations of TaAs-class topological Weyl semimetals under high pressure or extreme conditions.

## Results

**Figure 1:**
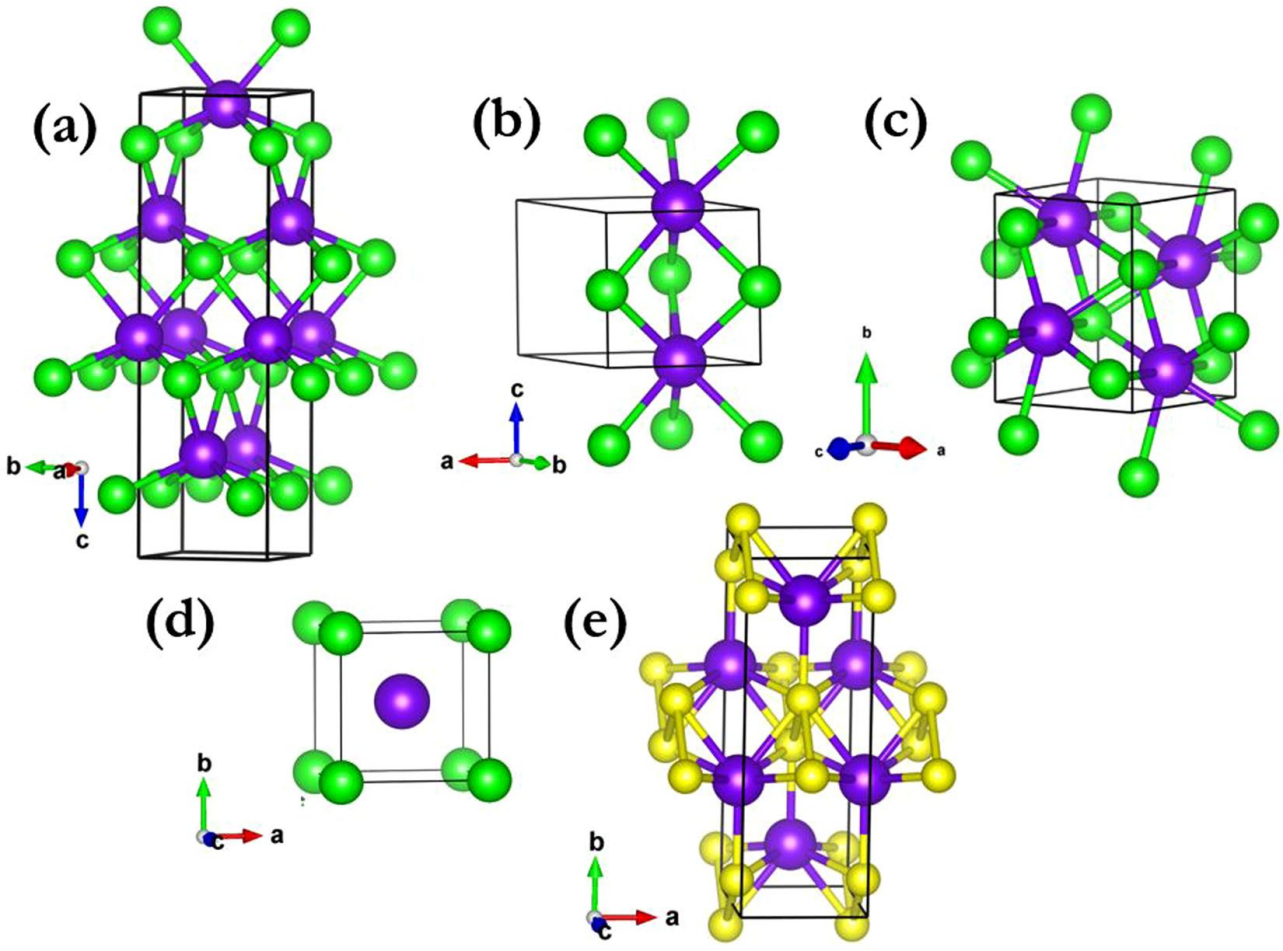
Crystal structures of NbAs and NbP, in which Nb, As, and P are represented as purple, green, and yellow balls, respectively. (**a**) *I*4_1_ *md* structure of NbAs at ambient pressure. (**b**) *P*-6m2 structure of NbAs at 30 GPa. (**c**) *P*2_1_/c structure of NbAs at 50 GPa. (**d**) *P*m-3m structure of NbAs at 80 GPa. (**e**) *C*mcm structure of NbP at 80 GPa.

**Figure 2:**
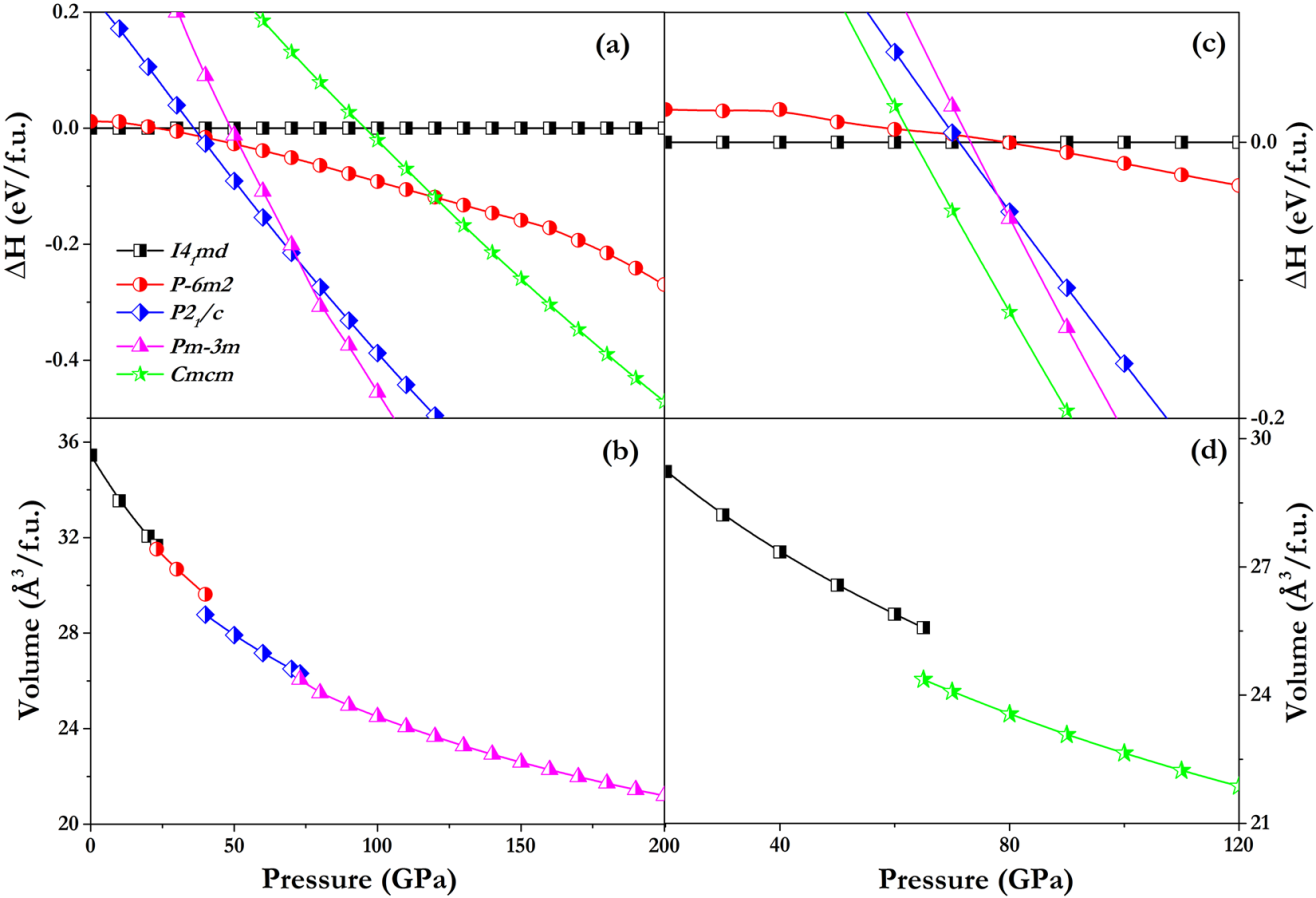
Calculated enthalpy difference per formula unit (f.u.) of different structures relative to the respective *I*4_1_ *md* ground-state structure and calculated volume per f.u. of the lowest-enthalpy phases as a function of pressure. **(a**,**b)** NbAs up to 200 GPa. **(c,d)** NbP up to 120 GPa for clarity.

TaAs-class Weyl semimetals, comprising TaAs^21^, TaP^22^, NbAs^23^, and NbP^24^, crystallize in a body-centred tetragonal symmetry with space group *I4*_*1*_ *md*, as shown in Fig. 1(a). The structure is distinguished by a lack of inversion symmetry, which is crucial for the Weyl semimetal state. To investigate the stable structures of NbAs and NbP under high pressure, all structures were fully geometrically optimized, allowing simultaneous variations of the unit cell and atomic positions at several pressures up to 200 GPa. The difference in enthalpy between the structures calculated at each pressure and the ground state structure is displayed in Fig. 2. The simulation identified the *I4*_*1*_ *md* structure as the ground state in both cases, which is consistent with experimental observations. High-pressure structure searches identified the sequential emergence of three structures (with *P-6m2*, *P2*_*1*_ */c*, and *Pm-3m* space groups) for NbAs, but only a *Cmcm* symmetry structure for NbP. To fully understand the phase transitions in NbAs and NbP, we also investigated the stability of *C*mcm structure of NbAs while *P-6m2*, *P2*_*1*_ */c*, and *Pm-3m* candidate structures of NbP since they share the same ambient structure. It is obvious to see that the NbAs with *Cmcm* symmetry is not energy favourable in the whole pressure from 0 GPa and 200 GPa, as illustrated in Fig. 2(a). Meanwhile, the *P-6m2*, *P2*_*1*_ */c*, and *Pm-3m* candidate structures of NbP have higher enthalpy as compared to the *Cmcm* structure from 63.5 GPa onwards, as displayed in Fig. 2(c). This further validates our structure search method adopted in this work.

The lattice constants and atomic coordinates optimized for the lowest-enthalpy structures at ambient conditions, together with previous experimental data^25^, are listed in Table 1. The deviation of the lattice constant is within 0.67% which is in good agreement with the experimental data. The detailed structural information in Table 1 is useful to the future XRD experimental refinements. The ground state *I4*_*1*_ *md* structure of NbAs remains stable until 23 GPa, beyond which a structure with *P-6m2* symmetry is favourable up to 38 GPa. Both phases incorporate NbAs_6_ polyhedra. It is interesting to note that the *P-6m2* phase has broken inversion symmetry and thus might be a Weyl semimetal. This possibility can be confirmed by further simulations of topological properties that are beyond the scope of our present study. At pressures beyond 38 GPa, a monoclinic *P2*_*1*_ */c* structure becomes the most energetically favourable phase and is predicted to be stable up to 73 GPa. Its crystal structure has inversion symmetry, and hence Weyl nodes are no longer preserved. Further compression induces a cubic phase with *Pm-3m* symmetry (with inversion symmetry) as most favourable from 73 to 200 GPa. These transition pressures are moderate and are accessible to the current experiment technique to further confirm our predictions. Combination of *in situ* Raman measurements with synchrotron XRD is commonly adopted and very effective to characterize the phase transformations behaviors under high pressure^26–28^. The coordination number of Nb increases from 6 (*P-6m2*) to 7 (*P2*_*1*_ */c*) and then to 8 (*Pm-3m*), as shown in Fig. 1, indicating a more dense packing under high pressure. TaAs has shown the same transition sequence under compression^29^. The volumetric changes are discontinuous: decreases of 0.5%, 2.9%, and 1.0% occur at 23, 40, and 73 GPa, respectively, as shown in Fig. 2(b). This is characteristic of first-order transitions. Interestingly, NbP shows only one transition, from the *I*4_1_ *md* ground state into a new structure with *Cmcm* symmetry at 63.5 GPa. This is accompanied by a significant volume drop of 4.7%, as shown in Fig. 2(c,d). This transition is distinct from those in TaAs and NbAs. The *Cmcm* structure possesses inversion symmetry, and thus cannot be a Weyl semimetal state.

**Table 1:** Calculated lattice constants and atomic positions for different phases of NbAs (*I*4_1_ *md*, *P*-6m2, *P*2_1_/c, *P*m-3m) and NbP (*I*4_1_ *md* and *C*mcm) at ambient pressure together with the experimental lattice constants of their *I*4_1_ *md* structures.

	Structure	Lattice constants (Å, degree)	Atomic positions
NbAs	*I4*_*1*_ *md*	a = b = 3.4726, c = 11.7569 (a = b = 3.452^24^ c = 11.679^24^)	Nb (0.0, 0.0, 0.04798), As (0.5, 0.5, 0.96614)
	*P*-6m2	a = b = 3.4253, c = 3.4863	Nb (0.33333, 0.66667, 0.0), As (0.0, 0.0, 0.5)
	*P*2_1_/c	a = 6.0999, b = 4.9330 c = 6.22, β = 132.0642	Nb (−0.67755, 0.64216, −0.30150), As (−0.20693, 0.65032, −0.27372)
	*P*m-3m	a = b = c = 3.2192	Nb (0.5, 0.5, 0.5), As (0.0, 0.0, 0.0)
NbP	*I4*_*1*_ *md*	a = b = 3.3441, c = 11.4315 (a = b = 3.334^24^, c = 11.376^24^)	Nb (0.5, 0.0, 0.27551), P (0.0, 0.5, 0.35813)
	*C*mcm	a = 3.1943, b = 8.916, c = 4.3175	Nb (0.0, 0.37343, 0.75), P (0.0, 0.09271, 0.75)

**Figure 3:**
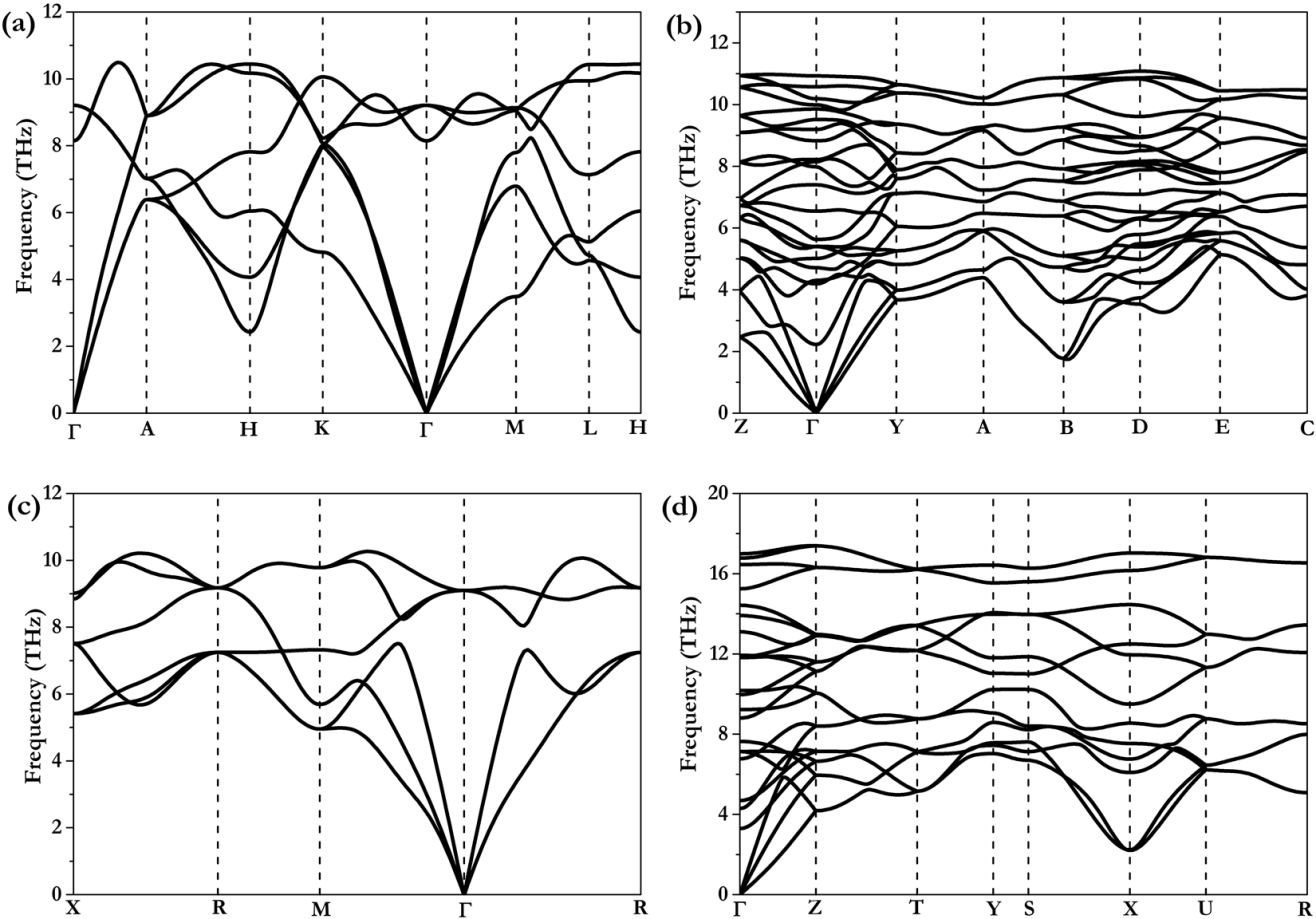
Phonon dispersions of NbAs and NbP. **(a)** *P*-6m2 structure of NbAs at 30 GPa, **(b)** *P*2_1_/c structure of NbAs at 50 GPa, **(c)** *P*m-3m structure of NbAs at 80 GPa, and **(d)** *C*mcm structure of NbP at 80 GPa.

To assess dynamic stability, phonon dispersion curves are calculated for the new high-pressure structures. No imaginary phonon frequencies are found in each stable pressure range in the whole Brillouin zone (Fig. 3), indicating the phases to be dynamically stable. Calculated phonon dispersion of these structures at ambient conditions shows that the frequencies all remain positive (data not shown). These new pressure-induced phases can be inferred to remain upon decompression to ambient conditions, thereby providing the opportunity to study high-pressure exotic behaviours at ambient pressure.

**Figure 4:**
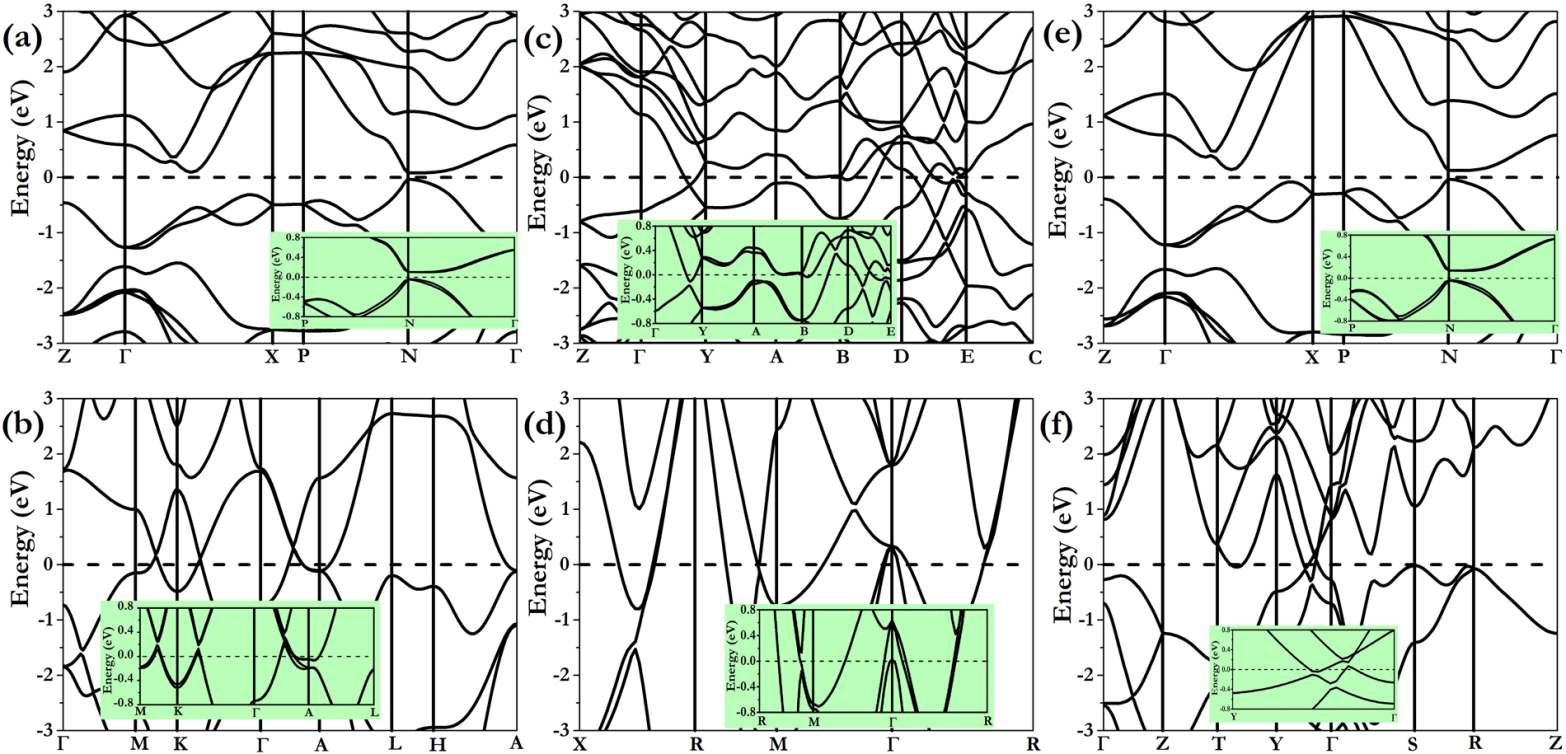
Calculated electronic band structures of NbAs and NbP at different pressures without spin–orbit coupling (SOC) while the insets including the SOC effect are enlarged along prominent direction. **(a)** *I*4_1_ *md* structure of NbAs at 0 GPa, **(b)** *P*-6m2 structure of NbAs at 30 GPa, **(c)** *P*2_1_/c structure of NbAs at 50 GPa, **(d)** *P*m-3m structure of NbAs at 80 GPa, **(e)** *I*4_1_ *md* structure of NbP at 0 GPa, and **(f)** *C*mcm structure of NbP at 80 GPa.

It is important to explore the evolution of the electronic structures of these compounds at high pressures. Calculated electronic band structures of NbAs (*I4*_*1*_ *md* at 0 GPa, *P-6m2* at 30 GPa, *P2*_*1*_ */c* at 50 GPa, and *Pm-3m* at 80 GPa) and NbP (*I4*_*1*_ *md* at 0 GPa and *Cmcm* at 80 GPa) with/without spin–orbit coupling (SOC) are shown in Fig. 4. Our results show the SOC effects give the splitting of the electronic bands degeneracies and change the crossing behaviour near the Fermi level. In the absence of SOC, NbAs shows a narrow bandgap of 0.11 eV around the *N* point while the SOC enhances the bandgap to 0.14 eV as illustrated in the Fig. 4(a) and its inset, indicating its semimetal character. It seems that previous crossing bands along K-Γ direction in Fig. 4(b) and crossing bands along Γ–Y direction together with D-E direction in Fig. 4(c) are fully gapped after taking SOC into account. Compression induces additional bands crossing the Fermi level and overlapping with each other, eventually leading NbAs to become a good metal with strong overlap between the conduction and valence bands in the *Pm-3m* phase at 80 GPa. The semimetal to metal transitions can be further confirmed through resistance measurements^20^. The electronic bands around Γ point change dramatically with the consideration of SOC, as indicated in Fig. 4(d). NbP is inferred to have a narrow bandgap of 0.14 eV without SOC (0.18 eV with SOC) that is also found at the *N* point of the Brillouin zone as shown in Fig. 4(e). With increasing pressure, the conduction band and valence band start to overlap along the Y–Γ direction, inducing a weak metallic nature. The SOC is found have a negligible influence on the band structure in the *Cmcm* phase of NbP, only slightly opens the gap around Y-Γ direction by 7 meV as shown in the Fig. 4(f).

**Figure 5:**
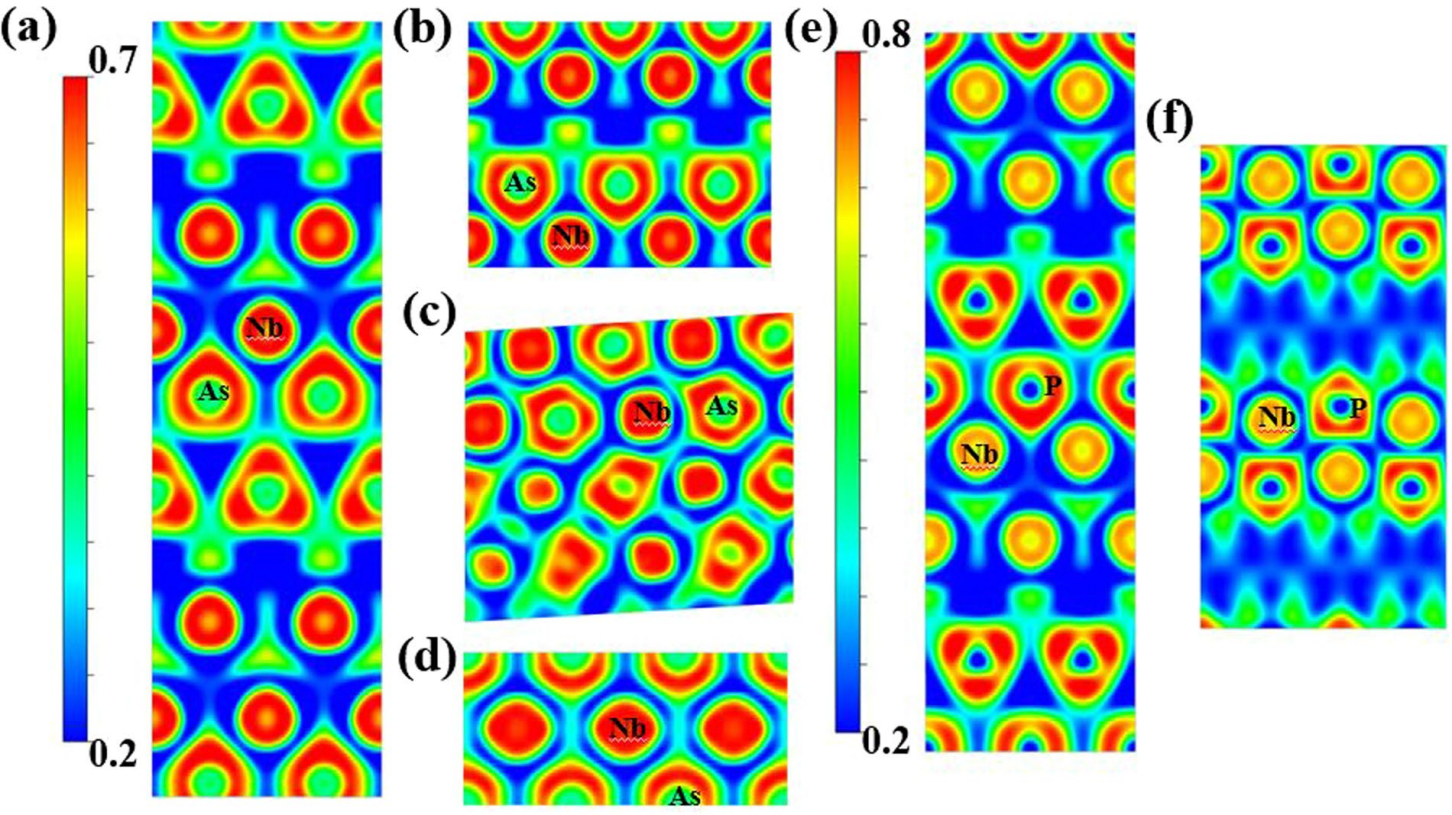
Calculated electron localization functions of NbAs and NbP at different pressures. **(a)** *I4*_*1*_ *md* structure of NbAs on the (100) surface at 0 GPa, **(b)** *P*-6m2 structure of NbAs on the  $(2\bar{1}0)$ surface at 30 GPa, **(c)** *P2*_*1*_ */c* structure of NbAs on the $(30\bar{4}1)$ surface at 50 GPa, **(d)** *P*m-3m structure of NbAs on the (110) surface at 80 GPa, **(e)** *I4*_*1*_ *md* structure of NbP on the (100) surface at 0 GPa, and **(f)** *C*mcm structure of NbP on the $(10\bar{1})$ surface at 80 GPa.

Electron localization function (ELF) simulation provides further understanding of high-pressure bonding behaviour. It can yield informative visual patterns of the core, binding, and lone-pair regions in chemical systems^30–32^. The results in Fig. 5 show electrons generally localized around the anions (As in NbAs and P in NbP) in all the phases. The most striking feature is the increasing localization with greater spherical symmetry around the anions in both systems as the pressure rises, indicating increasing iconicity bonding character. The charge transfer from As (P) to Nb is consistent with As (2.18) and P (2.19) being more electronegative than Nb (1.60). This pressure-induced charge transfer may also explain the phase transformations observed in NbAs and NbP.

## Conclusion

High-pressure structure prediction up to 200 GPa was conducted for Weyl semimetal (NbAs and NbP) using first-principles calculations combined with particle swarm optimization. NbAs showed a transition sequence of *I4*_*1*_ *md* → *P-6m2* → *P2*_*1*_ */c* → *Pm-3m* under compression with transition pressure around 23 GPa, 38 GPa, and 73 GPa, respectively. Remarkably, NbP showed a single *I4*_*1*_ *md* → *Cmcm* transition around 63.5 GPa. All these newly pressure-induced structures are dynamically stable at high pressure and at ambient conditions. Electronic band structure calculations demonstrated semimetal-to-metal transitions upon compression. The influence of the SOC on the electronic band structure was examined and SOC enhances the bandgap in *I4*_1_ *md* structure. Moreover, SOC effects are evidenced to give the splitting of the electronic bands degeneracies and change the crossing behaviour near the Fermi level. These results are meaningful and fundamental for future investigations of these TaAs-class Weyl semimetals under high pressure or extreme conditions.

## Methods

Extensive structure searching is carried out with the swarm intelligence CALYPSO method^33–36^, which enables global minimization of energy surfaces realized through first-principles total-energy calculations. This method has been successfully used to investigate high-pressure structures of various materials^37–42^. In sampling the energy surface, chemical composition is the exclusive input parameter. Structures searching here employs a system size ranging from 2 to 4 formula units per cell, and the pressure is limited to 200 GPa. We have run 50 structure generations and 1500 random structures were generated in total. 60% of the previous generation with lower Gibbs free energy are kept while the rest 40% of current generation are generated randomly which guarantees the diversity of the structure pool. A bond characterization matrix is employed as a fingerprint technique to remove the degenerate structures which enhances the search efficiency. The underlying structural relaxations and all the electronic structure calculations are performed in the framework of density functional theory using the generalized gradient approximation (Perdew–Burke–Ernzerhof functional)^43^, as implemented in the Vienna ab initio simulation package (VASP)^44^. Projector augmented wave potentials^45^ with 4s^2^4p^6^4d^4^5s^1^, 3s^2^3p^3^, and 3d^10^4s^2^4p^3^ valence electrons are adopted for Nb, P, and As, respectively. To ensure the calculated enthalpy converges well to better than 1 meV/atom, plane-wave kinetic energy cutoffs of 800 eV and a Monkhorst–Pack Brillouin zone sampling grid with a resolution of 2π × 0.02 Å^−1^ are chosen. The electronic band structure were calculated with and without the consideration of spin–orbital coupling (SOC) duo to its essential role for the studying intriguing topological electronic states^46^. Previous results show that the theoretical lattice parameters, and phonon frequencies are not sensitive to SOC effects^47^ and thus the SOC effects have been neglected to investigate the structural optimization and lattice dynamics. The dynamic stability of the predicted structures is determined by calculating the phonon dispersion using a supercell approach^48^, as implemented in the PHONOPY code^49^.
